# Ternary Logic of Motion to Resolve Kinematic Frictional Paradoxes

**DOI:** 10.3390/e21060620

**Published:** 2019-06-24

**Authors:** Michael Nosonovsky, Alexander D. Breki

**Affiliations:** 1 Mechanical Engineering, University of Wisconsin—Milwaukee, 3200 North Cramer St., Milwaukee, WI 53211, USA; 2 Department of Machine Design, St. Petersburg Polytechnic University, 29 Polytechnicheskaya St., 195251 St. Petersburg, Russia

**Keywords:** Painlevé paradoxes, friction, ternary logic, Łukasiewicz logic, entropic stability criteria, ultraslow friction

## Abstract

Paradoxes of dry friction were discovered by Painlevé in 1895 and caused a controversy on whether the Coulomb–Amontons laws of dry friction are compatible with the Newtonian mechanics of the rigid bodies. Various resolutions of the paradoxes have been suggested including the abandonment of the model of rigid bodies and modifications of the law of friction. For compliant (elastic) bodies, the Painlevé paradoxes may correspond to the friction-induced instabilities. Here we investigate another possibility to resolve the paradoxes: the introduction of the three-value logic. We interpret the three states of a frictional system as either rest-motion-paradox or as rest-stable motion-unstable motion depending on whether a rigid or compliant system is investigated. We further relate the ternary logic approach with the entropic stability criteria for a frictional system and with the study of ultraslow sliding friction (intermediate between the rest and motion or between stick and slip).

## 1. Introduction

Dry sliding friction is a universal phenomenon observed at the scales ranging from nano-newtons to billions of tons for all classes of materials (polymers, metals, ceramics, composites, etc.) and their combinations. However, the way of how friction is treated in physics is far from general or universal. The so-called Amontons–Coulomb laws of dry friction were formulated by the 18th century, and they describe a theory which is referred to as “the Coulomb friction.” These laws state that the friction force between two contacting bodies, *F*, is independent of the nominal area of contact, sliding velocity, and that *F* is linearly proportional to the normal force, *W*, acting between the two bodies. In the form of an equation this is formulated as

*F* = *μW*, (1)

where *μ* is the coefficient of friction (COF), which is independent of the normal force, sliding velocity, and the nominal area of contact between the bodies. 

The physical foundations of the Coulomb friction theory have been discussed and criticized in literature [1], while various modifications of the friction laws have been suggested including, for instance, the rate-and-state models with internal variables [2] or the “friction is fracture” concept [3]. However, the Coulomb law in the form of Equation (1) and its version for the normal and shear stress components, *τ*_xy_ = *μσ*_yy_, still remain the most common way to introduce friction into mechanical analysis of both rigid and deformable systems. 

Usually, the Amontons–Coulomb laws of dry friction are introduced in an ad hoc manner, without any justification from the deeper principles of physics, although some attempts to either derive the laws of friction from more fundamental principles, such as the second law of thermodynamics or principles of brittle fracture are sometimes introduced. However, in most textbooks the Amontons–Coulomb laws are treated as approximate empirical rules rather than exact laws of nature. 

Due to this ambiguous status of friction in physics—universal on the one hand and unrelated to the fundamental principles of physics on the other hand—and despite the successful application of the Amontons–Coulomb friction laws, combining friction with the rest of mechanics and physics involves a number of interesting logical paradoxes. The best-known family of frictional paradoxes are the Painlevé paradoxes involving rigid (non-deformable) bodies [4,5,6,7,8,9,10,11]. When a solution of equations of motion with friction is obtained, it may turn out that the assumed direction of the friction force contradicts the direction of the velocities in the system, therefore resulting in a paradox. The Painlevé paradox indicates that the Coulomb friction is not always logically compatible with the rest of the equations of mechanics. The differential equations of motion in mechanics are supposed to have a unique solution corresponding to any reasonable set of initial conditions. However, a system with the paradox either has no solution or it has more than one solution. 

The original 1885 publication by Painlevé [12] caused a long discussion, in which participated prominent physicists and mechanicians such as L. Prandtl, F. Klein, R. Von Mises, and Lecornu. L. Lecornu [13,14] suggested abandoning the model of the absolute rigid body, while F. Klein [15] insisted that the Coulomb friction is not in contradiction neither with principles of mechanics nor with the phenomena observable in nature, and Von Mises stated that the methodology of Newtonian mechanics compels us to modify the Coulomb law [16]. 

There is significant literature about the nature of the Painlevé paradox and various approaches to resolve it [17,18]. These approaches typically modify the law of friction [19] by suggesting a non-Coulombian friction law, e.g., velocity dependent [4], or they consider deformable bodies instead of rigid ones [5,6,7]. Some approaches concentrate on computational schemes, including the use of Convex Analysis [8] and the variational inequality approaches [9]. The relationship of the Painlevé paradoxes to other mathematical problems, such as the linear complementary problem, has also been investigated [10], as well as the relationship between the paradoxes to the friction-induced dynamic instabilities and self-excited vibrations [11,20]. 

Besides the Painlevé paradoxes, there are several other types of frictional paradoxes [21,22]. Some of these paradoxes emerge because in general the introduction of the compliance does not resolve the Painlevé paradoxes. Dynamic effects in elastically deformable systems lead to different types of frictional paradoxes with the assumed direction of sliding used for the Coulomb friction opposite to the resulting slip velocity [21]. This suggests that the Coulomb friction is inconsistent not only with the rigid body dynamics, but also with the dynamics of the elastically deformable bodies.

In a formal logical sense, there are several ways of resolving logical paradoxes. One method is to introduce a multi-valued logic, in which the law of excluded middle does not apply, and unlike in the regular binary logic, logical variables can attain values beyond *true* and *false*. Note that different interpretations of the third truth value have been suggested including *unknown*, *possible*, *undefined*, *half-true*, *irrelevant*, and *inconsistent* [23]. Despite that, ternary logic has been applied successfully to various problems ranging from computer science to oscillating chemical reactions [24]. The idea that some parts of mechanics can be represented by means of pure symbolic logic has also been suggested, for example, by A. Zinov’ev [25]. However, nowadays this idea is often connected to the paradigm of unconventional computing, according to which different natural systems have their different own symbolic logics [26,27]. Therefore, a three-valued symbolic logic can also be applied to mechanical problems with friction. 

In this paper, we will discuss the application of ternary logic to the frictional paradoxes, its productivity for the analysis of mechanical problems, and how it can affect the fundamental mechanical concepts of “motion”, “rest”, and “stability”.

## 2. Examples of Frictional Paradoxes

**Figure 1:**
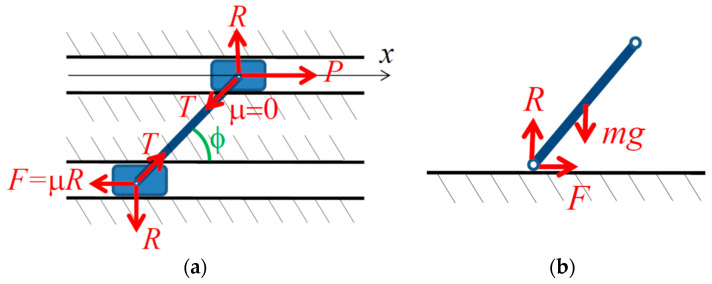
Setup for the Painlevé paradox: (**a**) two sliders (one frictional and the other is frictionless) connected by a rigid bar; (**b**) rigid slender rod falling under gravity.

An example is in Figure 1a, showing two sliders of equal mass *m* connected by a rigid link with a constant length *l* at an angle φ with the sliding surface. The upper slider is frictionless while the lower slider is frictional with the COF *μ*. An external positive force *P* is applied to the upper slider. The normal force *R* acts at the first slider, so that *T* = *R/*sinφ is the tension/compression force in the link, and $F=\mu \left|R\right|\mathrm{sign}\left(\dot{x}\right)$ is the friction force on the second slider. The motion of this system is governed by the equation

(2) $$2m\ddot{x}=P-\mu \left|R\right|\mathrm{sign}\left(\dot{x}\right)$$

with two unknowns, $\ddot{x}$ and *R*, related by the equation of motion for the upper body, $m\ddot{x}=P-R/\tan\left(\varphi \right)$. Note that for *P* > 0, μ>0, tanφ>0, and the sign of *R* depends on the sign of $P-m\ddot{x}$, In order to solve Equation (2), one should assume a value of $\mathrm{sign}\left(\dot{x}\right)$. Substituting $R=\left(P-m\ddot{x}\right)\tan\left(\varphi \right)$ into Equation (2) and we obtain four possible cases:

(a) If $P\geq m\ddot{x}$ and $\dot{x}>0$ then $m\ddot{x}=P\frac{1-\mu \tan\varphi }{2-\mu \tan\varphi }$, which is possible only for μtanφ<2 (otherwise $P<m\ddot{x}$).
(b) If $P\geq m\ddot{x}$ and $\dot{x}<0$ then $m\ddot{x}=P\frac{1+\mu \tan\varphi }{2+\mu \tan\varphi }$.
(c) If $P<m\ddot{x}$ and $\dot{x}>0$ then $m\ddot{x}=P\frac{1+\mu \tan\varphi }{2+\mu \tan\varphi }$, which contradicts $P<m\ddot{x}$.
(d) If $P<m\ddot{x}$ and $\dot{x}<0$ then $m\ddot{x}=P\frac{1-\mu \tan\varphi }{2-\mu \tan\varphi }$, which is possible only for μtanφ>2.

Therefore, two solutions (cases b and d) exist when $\dot{x}<0$ and μtanφ>2, one solution (case b) when $\dot{x}<0$ and μtanφ<2, one solution (case a) when $\dot{x}>0$ and μtanφ<2, and no solution when $\dot{x}>0$ and μtanφ>2.

A slightly different version of the paradox, close to the original problem studied by Painlevé in 1905 [28], involves a rigid slender rod of mass *m* in contact with a rigid surface and falling under gravity (Figure 1b).

## 3. Ternary Logic of Motion and Rest

The Painlevé paradox indicates that the Coulomb friction is not always logically compatible with the rest of the equations of mechanics. To express this situation in a formal manner, we can introduce a logical predicate *P*(*m*, *F*, *μ*, φ) defined over a system of mechanical equations (Equation (2)) [11]. The predicate can attain two values, *P* = *true*, when the system is at rest, or *P* = *false* when the system is in motion.

In a regular mechanical system without friction, there are only two possible states of the system: it is either at rest or in motion, or

(3) ∀(m, F, μ,φ)P⊕¬P=true,

where ⊕ stands for the exclusive disjunction (“exclusive or”) operator and ¬ is the negation. However, in a system with paradoxes, the classical logical law of the excluded middle (*tertium non datur*) of the binary Aristotelean logic is not valid anymore. Besides the state of the rest and the state of the motion, there are values of the parameters *m*, *F*, *μ*, and φ which correspond to the paradox. In the context of friction, the states of rest and motion are typically called the *slip* and *stick* states.

For a system with the Coulomb friction, such as in Figure 1, the solution exists when and only when parameters are within the specified range, for example

(4) (μtanφ≤2)⇔(P(m, F, μ,φ)⊕¬P(m, F, μ,φ))

which is equivalent to

(5) (μtanφ>2)⇔(P(m, F, μ,φ)∧¬P(m, F, μ,φ))

(6) (μtanφ>2)⇔(P(m, F, μ,φ)=undefined)

To accommodate the undefined state, we will use the following relationships of the Łukasiewicz logic [29].

We suggest the following physical interpretation of these logical operations. The value *P* = *true* is the rest, *P* = *false* is the motion, and *P* = *undefined* is the paradox. The Conjunction (Table 1) corresponds to a situation, when a larger system consists of several parts. Only if all parts of a system are at rest, the entire system will be at rest. If at least one part of the system is in motion, then the entire system is in motion. If some parts of the system are at rest, while others have a paradox, the entire system is in paradox. If some parts of the system are in motion, while others have a paradox, the entire system is in motion. 

The Disjunction (Table 2) can be defined as a situation when at least one part of the system has a part in a given state. If one part of the system is at rest and another is in motion or has a paradox, then a resting part is present. If one part of the system is in motion, and another has a paradox, then a paradoxical part is present. 

Negation rules (Table 3) in the Łukasiewicz logic imply an opposite state: a resting system is opposite to a moving system, while the undefined state is opposite to itself. Other logical functions, such as the exclusive or and the Implication, can be defined as well.

## 4. Implications to the Stability

### 4.1. Resolution of Paradoxes by Introducing Compliance

**Figure 2:**
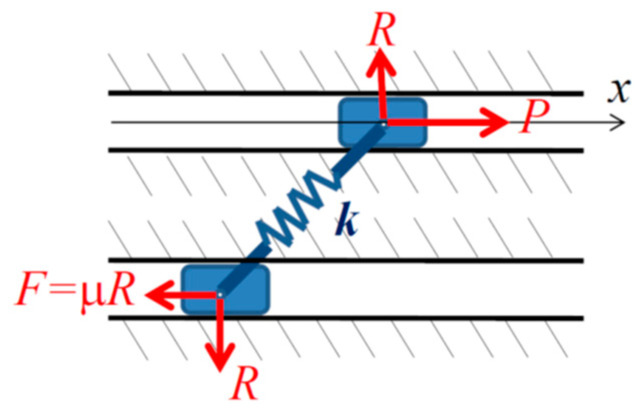
The paradox is resolved if the bar is assumed to be elastically deformable with the compliance *k*.

In order to introduce ternary logic into the mechanics of frictional motion, one should demonstrate that ternary logic is productive for problems of classical mechanics. To generalize the ternary logic approach, we will consider the relation between the Painlevé paradoxes in a non-deformable system and the stability of a compliant system. The introduction of a compliant link instead of a rigid one often resolves a Painlevé paradox; however, the paradox in a non-deformable system can correspond to instability in a compliant system (Figure 2), as discussed in references [5,6]. If an elastically deformable link with the compliance *k* (the inverse of the stiffness or of the elastic modulus) is considered instead of the rigid link, the sliding system obtains an additional degree of freedom. In that case, the paradox corresponds to the dynamically unstable solution with the reaction force growing until the value of φ decreases so that the paradox condition μtanφ>2 will not be satisfied anymore. Thus the paradox of a non-existent solution, when studied within the dynamics of deformable bodies, corresponds to an unstable solution.

A predicate S(*α*, *β*, …) meaning “the motion/equilibrium is dynamically stable” can be introduced. Note that the predicate is binary, i.e., it can only attain values S = *true* and S = *false*. A system consisting of two parts, *A* and *B*, is stable only when both are stable: S(A∧B)=S(A)∧S(B) [11].

For the system in Figure 1, the predicate is defined according to the stability condition;

(7) ((k≠0)∧( μtanφ≤2))⇒S(μ, φ, k)

(8) ((k≠0)∧( μtanφ>2))⇒¬S(μ, φ, k)

Equations (7) and (8) are parallel to the condition of the existence of the paradox in the rigid system:

(9) ((k=0)∧(μtanφ≤2))⇒(P(μ, φ,k)⊕¬P(μ, φ,k))

(10) ((k=0)∧( μtanφ>2))⇒(P(μ, φ,k)=undefined)

It is concluded from Equations (7)–(10) that in the limit of small compliance, k→0, when the deformable link becomes a rigid one, the unstable motion becomes equivalent to the paradox (in the binary logic) or to the undefined state of the predicate P:

(11) ¬S(μ, φ, k)⇔(P(μ, φ,k)=undefined), for k=0.

In other words, the ternary logic of the rigid (*k* = 0) system with predicate’s values corresponding to “the system is at rest/moving/undefined” obtains a new interpretation for compliant (k≠0) systems: “at rest/stable motion/unstable.”

Considering the *unstable motion* as a third possibility besides the *rest* and the *stable motion* is very productive. The stability of a system is now defined as S(A)=P(A)∨¬P(A) (“the system either at rest or stable motion”). The stability of a combined system, *A* and *B*, is then defined as S(A∧B)=(P(A)∨¬P(A))∧(P(B)∨¬P(B)), or S(A∧B)=(P(A)∧P(B)) ∨(P(A)∧¬P(B))∨(¬P(A)∧P(B))∨(¬P(A)∧¬P(B)), which is equivalent to ¬S(A∧B)=((P(A)=undefined)∨(P(B)=undefined)). Thus if any part of a system is unstable (neither in rest nor in stable motion), then the entire system is unstable.

### 4.2. Friction-Induced Instabilities and Entropic Stability Criterion

**Figure 3:**
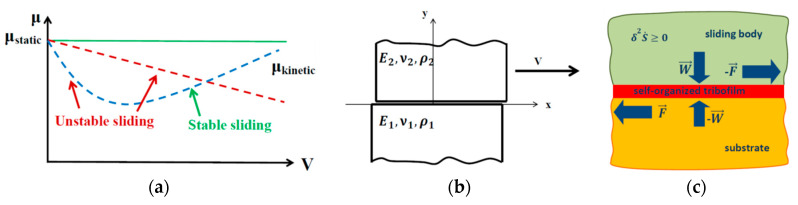
(**a**) Three possible dependencies of the coefficient of friction (COF) on sliding velocity (constant COF, decreasing COF, and COF with a minimum value). Decreasing COF can cause the instability; (**b**) two elastic half-spaces (characterized by the elastic moduli, *E*_1_ and *E*_2_, Poison’s ratios *ν*_1_ and *ν*_2_ and densities ρ_1_ and ρ_2_) slide relative to each other with the velocity *V*. For slightly dissimilar materials (in terms of their elastic properties) an interface elastic wave can propagate, and the wave becomes unstable when friction is introduced. (**c**) Schematic of friction induced self-organization of a tribofilm (based on discussion in Reference [20]).

Although friction is usually considered as a stabilizing factor, sometimes introducing friction at the sliding interface leads to the dynamic instability. A simple example is when the COF decreases with increasing sliding velocity (Figure 3a). A small increase of the velocity due to a fluctuation results in the decrease of frictional resistance and in further increasing velocity, hence the instability. More interesting cases of frictional instabilities are found when no velocity dependency exists, but other factors, such as the elasticity of the material, are introduced. Thus, for two smooth elastic bodies sliding with friction relative to each other, a small disturbance can grow exponentially with time (Figure 3b). Mathematically, the stability analysis of sliding of two elastic half-spaces having a constant coefficient of friction between them is a relatively simple mathematical problem. However, it had not been studied until the 1990s, when Adams [30] discovered that the motion is dynamically unstable for a broad range of material parameters. Without friction, an elastic wave can propagate along the interface between two elastic bodies. This wave is confined to the interfacial area, because the wave magnitude decreases exponentially with the distance from the interface. These interfacial waves are often called *generalized Rayleigh waves* (GRWs), because they generalize the concept of the elastic surface wave, referred to as the *Rayleigh wave*. 

When a small constant COF is introduced, the amplitude of the GRW will not remain constant anymore. Instead, the amplitude will grow exponentially with time thus making the sliding dynamically unstable. The source of energy for these growing-amplitude waves is in the work done by the external force applied to overcome friction. A similar effect was found for rough surfaces as well [31]. 

Different types of instabilities emerge when the COF is coupled with another effect, such as the thermal expansion of the material. A general thermodynamic stability criterion has been suggested to study any type of instabilities [20,32]. The criterion states that the second variation of the rate of entropy (*s*) production per unit time should be positive, $\delta^{2}\dot{s}>0$, in order for the motion to remain stable [32]. The expression for the entropy rate can include mechanical, thermal, chemical, electric, micro/nanostructural, and other components. 

If only mechanical interactions occur in a frictional system with one degree of freedom, then the rate of entropy is given by the rate of energy dissipation (the product of the sliding velocity *V*, and the friction force *μW*) divided by temperature, $\dot{s}=\mu WV/T$. If two of these parameters are varied and interrelated, we obtain

(12) $$\delta^{2}\dot{s}=\frac{W}{T}\delta V\delta \mu =\frac{W}{T}\frac{d\mu }{dV}{\left(\delta V\right)}^{2}>0\;\mathrm{or}\;\frac{d\mu }{dV}>0$$

Using the stability logical predicate *S* as introduced in the preceding section, one can define

(13) $$S\equiv \left(\delta^{2}\dot{s}>0\right)$$

Note that the stability S(δV, δμ) depends on the *variations* of the COF and sliding velocities, rather than on the values of these parameters. One can write then a formal proof

(14) $$\frac{d\mu }{dV}>0\Rightarrow \frac{\delta \mu }{\delta V}>0\Rightarrow \frac{W}{T}\delta V\delta \mu >0\Rightarrow \delta^{2}\dot{s}>0\Rightarrow S\left(\delta V,\;\delta \mu \right)$$

In other words, if the COF decreases with the sliding velocity, the system is unstable. This is because an increasing sliding velocity causes a decrease in the frictional resistance and further increase of the velocity. 

The interactions, which occur when frictional sliding is coupled with another process that contributes to entropy production, such as thermally activated material transfer, chemical reactions, or wear [20], are much more interesting and complex than pure mechanical interactions. 

For example, the COF may depend, instead of the sliding velocity, on the thickness of the in situ formed interfacial film in a composite material or in a metallic alloy (Figure 3c). The increased thickness of the film may result in decreased friction, forming a feedback loop “friction–film thickness” similar to the “friction–sliding velocity” loop described by Equation (14). Consequently, a protective self-lubricating tribofilm can form at the frictional interface under favorable conditions (the best-known example is a copper film formed at the bronze-steel frictional interface) [20].

## 5. Discussion

Besides its importance to the resolution of frictional paradoxes, the logical juxtaposition of the three types of solutions of mechanical equations—rest, stable motion, and unstable motion—has interesting parallels in the history of mechanics and it has relevance to emerging new areas. 

### 5.1. Note on the History of Mechanics

Throughout the history of pre-modern mechanics, the state of *rest* and the state of *motion* were considered two opposite states of a mechanical system, rather than *rest* being a special case of *motion*. This is because in Aristotle’s physics, no motion by inertia was possible, and motion always implied the presence of a moving force or an effective cause of motion [33]. 

Several paradoxes have emerged accompanying the opposition of *rest* vs. *motion*, including the classical Zeno’s arrow paradox, formulated by Aristotle as “If everything when it occupies an equal space is at rest, and if that which is in locomotion is always occupying such a space at any moment, the flying arrow is therefore motionless.” [25,33] 

Furthermore, it was a matter of discussion, whether rest can be obtained as a combination of two uniform motions, until the so-called Tusi couple was discovered in the 13th century. The Tusi couple is an imaginary device used for the Copernican astronomical model. The Tusi couple consists of two spheres with a smaller sphere rolling inside a larger sphere having twice the same diameter. A point on a smaller sphere performs an oscillatory motion. At an extreme point of its trajectory, the point changes its direction for an opposite one, so that the instantaneous velocity is zero. While the mechanism is similar to the crank-slider linkage, which converts rotation into the reciprocating motion, and it was known from ancient times, the Tusi couple demonstrated that continuous rotation can produce motion with instantaneous zero velocity, which was not obvious until the concept of instantaneous velocity was suggested [34].

It took significant efforts, until the motion by inertia (without cause) was discovered by Galileo in the early 1600s (in fact, Giuseppe Moletti had already established that objects of different weight fall with the same acceleration [35]). This required the realization that friction is what prevents moving objects from continuous motion by inertia. Therefore, friction and inertia were in a complimentary relationship: without identifying friction, inertia could not be recognized. 

H.A. Wiltsche pointed out that pre-Galilean Aristotelian mechanics studied natural *occurrences* as opposed to the study of *phenomena* (“the invariant forms that allegedly underline natural occurrences”) introduced by Galileo. The latter systematically excluded causal accidents as impediments, and friction largely fell as a victim in the search of refined and purified phenomena [36]. Even today, despite almost universal occurrence of friction, it is studied by materials scientists and engineers much more often than by physicists. 

After calculus was also created in the 17th century by Newton and Leibnitz, it became possible to introduce the concept of instantaneous velocity as a derivative of coordinate, and consider rest as a special zero-velocity case of motion. Since then, it has been established that the positions and velocities corresponding to degrees of freedom of a mechanical system characterize a state of that system. The approach was extended in a formal way by the Lagrange mechanics, which views the law of motion as a local extremum of a functional depending on the generalized coordinates in the configurational space of the system, and further by the Hamilton mechanics, which defines the law of motion in the phase space of a mechanical system (a cotangential bundle of the configurational space) given by coordinates and momenta.

### 5.2. Ultraslow Frictional Sliding between Motion and Rest

**Figure 4:**
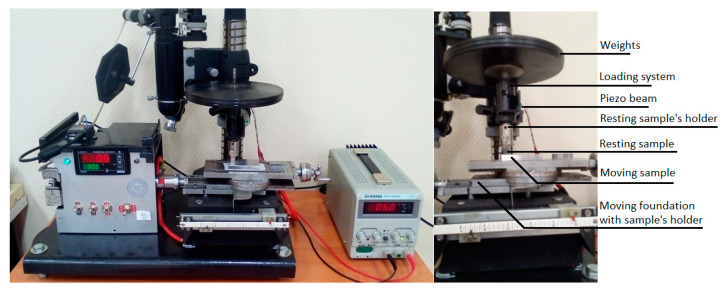
The ultraslow sliding friction tribometer MTBM: a general view and the setup (Reproduced from [37], with the permission of AIP Publishing).

While most traditional mechanical systems possess a clear distinction between the states of motion and rest, some systems can be characterized by an intermediate state between motion and rest, including the so-called *ultraslow* or *super-slow* motion. Ultraslow processes are defined as processes with the rate of change of the parameters comparable or smaller than the fluctuations of their measurements. An example is supplied by ultra-slow frictional sliding, which is defined as sliding with the velocity comparable with the relaxation rate of the processes in the material [37]. Practically speaking, this is a nanovelocity, typically in the range of nanometers per second (Figure 4).

**Figure 5:**
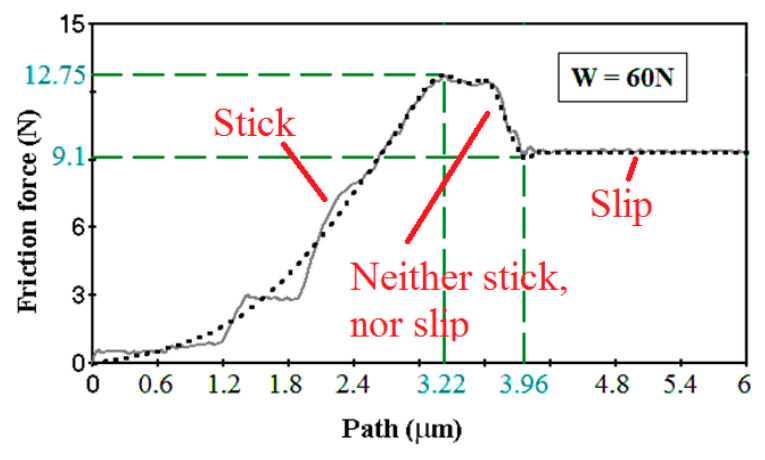
A typical dependency of the friction force on the frictional path for the ultraslow sliding (37 nm/s, normal load *W* = 60 N). The friction force drops steadily from the static friction value (*F* = 12.75 N) to the kinetic friction value (*F* = 9.1 N) thus demonstrating intermediate values between stick and slip. Based on results discussed in [37].

In the context of friction, motion and rest are often called *slip* and *stick*, while the static COF in the stick state is typically larger than the kinetic COF in the slip state. The ultraslow sliding friction is, in a sense, between the static friction and kinetic friction. For friction between two very smooth steel samples (so-called Jo blocks) at the sliding velocity of 37 nm/s, the force-displacement dependencies suggest that the onset of sliding is a gradual transition between two regimes rather than an abrupt irreversible transition from static to kinetic friction observed at higher sliding velocities (Figure 5). Thus, ultraslow sliding can be interpreted as a state which is neither stick nor slip.

### 5.3. Mechanics of Instabilities

Traditionally, mechanics studies stable processes and stable solutions of the equations of motion, with unstable solutions considered non-physical and thus irrelevant. However, unstable processes and regimes of motion play a role in various situations, from flight aerodynamics (flying objects, from insects to fighter jets, may have unstable phases of motion when they maneuver) to rapid processes such as explosions or crack propagations during fracture. Non-stable solutions are usually just sorted out and not considered. While unstable processes are qualitatively different from stable processes, traditional mechanics has limited tools to adequately reflect this difference. The proposed logical formalism can fill this gap. 

Investigating unstable motion introduces a new and diverse class of mechanical phenomena, which have rarely been investigated by mechanicians until the end of the 20th century, including those leading to the self-organization and hierarchical structures. 

The advances of recent decades in studying non-equilibrium processes made it clear that “unstable motion” is a very important area of research and that it can lead to engineering applications, such as the development of new materials. Note that frictional systems can lead to new logical and informational devices, if friction is viewed from the viewpoint of information production, rather than from the view point of energy dissipation [11].

## 6. Conclusion

In the classical Newtonian and Lagrangian mechanics of particles, a state of the system with *N* degrees of freedom is characterized by a set of coordinates and velocities $\left(x_{n},{\dot{x}}_{n}\right)$, n=1, …,N. We have suggested an extension of this description, $\left(x_{n},{\dot{x}}_{n},P_{n}\right)$, where $P_{n}\equiv \left({\dot{x}}_{n}=0\right)$ is a logical variable, which may attain three values: *true* (the system at rest), *false* (the system in motion, which is assumed stable), and *undefined*. The undefined state can be interpreted as a paradoxical situation (when no solution or non-unique solution exists), as an instability, or as an intermediate state between rest and motion in a certain sense (e.g., the ultraslow sliding). The overall state of the system is given by the conjunction of the degrees of freedom $P=P_{1}\wedge P_{2}\wedge \ldots \wedge P_{N}$. Thus, the system is at rest if all velocities are defined and equal to zero, the system is moving if at least one velocity is defined and non-zero, and the system is undefined otherwise. For example, the stability of the system can be defined as $S=S_{1}\wedge S_{2}\wedge \ldots \wedge S_{N}$, where $S_{n}=P_{n}\vee \neg P_{n}$. 

Certainly, the actual behavior of a mechanical system does not depend on the logical apparatus used. The paradoxes are related to shortcomings of the models, which describe mechanical systems with friction. The main advantage of the proposed three-valued logical description is that it formally addresses and often resolves frictional paradoxes in mechanical models (when no solution exists or a solution is non-unique) in a natural way, and allows to clearly distinguish between stable motion, unstable solutions, and rest as qualitatively different states of a mechanical system. The proposed method can be applied to the analysis of unstable motion and to the analysis of situations, when intermediate states between rest and motion exist, such as the ultraslow motion.

## Figures and Tables

**Table 1:** Conjunction (A∧B).

A∧B		B		
		False	Undefined	True
A	False	False	False	False
	Undefined	False	Undefined	Undefined
	True	False	Undefined	True

**Table 2:** Disjunction (A⋁B).

A⋁B		B		
		False	Undefined	True
A	False	False	Undefined	True
	Undefined	Undefined	Undefined	True
	True	True	True	True

**Table 3:** Negation (¬A).

A	¬A
False	True
Undefined	Undefined
True	False
